# Engineering a natural *Saccharomyces cerevisiae* strain for ethanol production from inulin by consolidated bioprocessing

**DOI:** 10.1186/s13068-016-0511-4

**Published:** 2016-04-30

**Authors:** Da Wang, Fu-Li Li, Shi-An Wang

**Affiliations:** Shandong Provincial Key Laboratory of Synthetic Biology, Qingdao Institute of Bioenergy and Bioprocess Technology, Chinese Academy of Sciences, Qingdao, 266101 China; University of Chinese Academy of Sciences, Beijing, 100039 China

**Keywords:** Yeast, Inulin, Ethanol, Ploidy, Consolidated bioprocessing, Protein secretion

## Abstract

**Background:**

The yeast *Saccharomyces cerevisiae* is an important eukaryotic workhorse in traditional and modern biotechnology. At present, only a few *S. cerevisiae* strains have been extensively used as engineering hosts. Recently, an astonishing genotypic and phenotypic diversity of *S. cerevisiae* was disclosed in natural populations. We suppose that some natural strains can be recruited as superior host candidates in bioengineering. This study engineered a natural *S. cerevisiae* strain with advantages in inulin utilization to produce ethanol from inulin resources by consolidated bioprocess. Rational engineering strategies were employed, including secretive co-expression of heterologous exo- and endo-inulinases, repression of a protease, and switch between haploid and diploid strains.

**Results:**

Results from co-expressing endo- and exo-inulinase genes showed that the extracellular inulinase activity increased 20 to 30-fold in engineered *S. cerevisiae* strains. Repression of the protease PEP4 influenced cell physiology in late stationary phase. Comparison between haploid and diploid engineered strains indicated that diploid strains were superior to haploid strains in ethanol production albeit not in production and secretion of inulinases. Ethanol fermentation from both inulin and Jerusalem artichoke tuber powder was dramatically improved in most engineered strains. Ethanol yield achieved in the ultimate diploid strain JZD-InuMKCP was close to the theoretical maximum. Productivity achieved in the strain JZD-InuMKCP reached to 2.44 and 3.13 g/L/h in fermentation from 200 g/L inulin and 250 g/L raw Jerusalem artichoke tuber powder, respectively. To our knowledge, these are the highest productivities reported up to now in ethanol fermentation from inulin resources.

**Conclusions:**

Although model *S. cerevisiae* strains are preferentially used as hosts in bioengineering, some natural strains do have specific excellent properties. This study successfully engineered a natural *S. cerevisiae* strain for efficient ethanol production from inulin resources by consolidated bioprocess, which indicated the feasibility of natural strains used as bioengineering hosts. This study also presented different properties in enzyme secretion and ethanol fermentation between haploid and diploid engineering strains. These findings provided guidelines for host selection in bioengineering.

**Electronic supplementary material:**

The online version of this article (doi:10.1186/s13068-016-0511-4) contains supplementary material, which is available to authorized users.

## Background

The budding yeast *Saccharomyces cerevisiae* is an important cell factory for production of traditional foods, enzymes, and pharmaceuticals. Recently, this yeast has been employed as a bioengineering platform for production of commodity chemicals, biofuels, and natural products [1–3]. At present, only a few strains have been extensively used as engineering hosts, such as strains from CEN.PK family and S288C series [4–7]. Recent ecology studies on *S. cerevisiae* identified a great diversity of natural isolates and recognized 13 clean lineages from various ecological sources and geographical locations [8, 9]. Interestingly, there are natural strains that can outcompete industrial strains in stress tolerance and such strains may be good candidate hosts for bioethanol production from sustainable biomass resources [10–12].

Bioethanol are mainly produced from the carbohydrates starch and sucrose conserved in arable-land-based plants, while sustainable sugars have attracted increasing attention in recent years, such as cellulose, alginate, mannitol, and inulin [13–16]. Inulin is a naturally occurring storage polysaccharide present in numerous plants, such as Jerusalem artichoke and chicory. The plant Jerusalem artichoke can grow well in poor soil without competition with food plants for farmland. Recently, Jerusalem artichoke is treated as a sustainable feedstock for bioethanol production by consolidated bioprocess (CBP). CBP requires yeasts to complete the processes of inulinase production, inulin hydrolysis, and fermentation in one reactor [10, 17]. Inulin consists of linear chains of β-2,1-linked D-fructofuranose molecules terminated by a glucose residue and it can be hydrolyzed into fermentable monosaccharide sugars fructose and glucose by inulinases. Inulinases are synthesized in a variety of microbes but not in *S. cerevisiae*. Although *S. cerevisiae* has an invertase SUC2 that possesses exo-inulinase activity, the yeast cannot efficiently convert inulin into ethanol because of the low activity of SUC2 toward inulin molecules with high degree of polymerization (DP) values [18, 19].

**Fig. 1:**
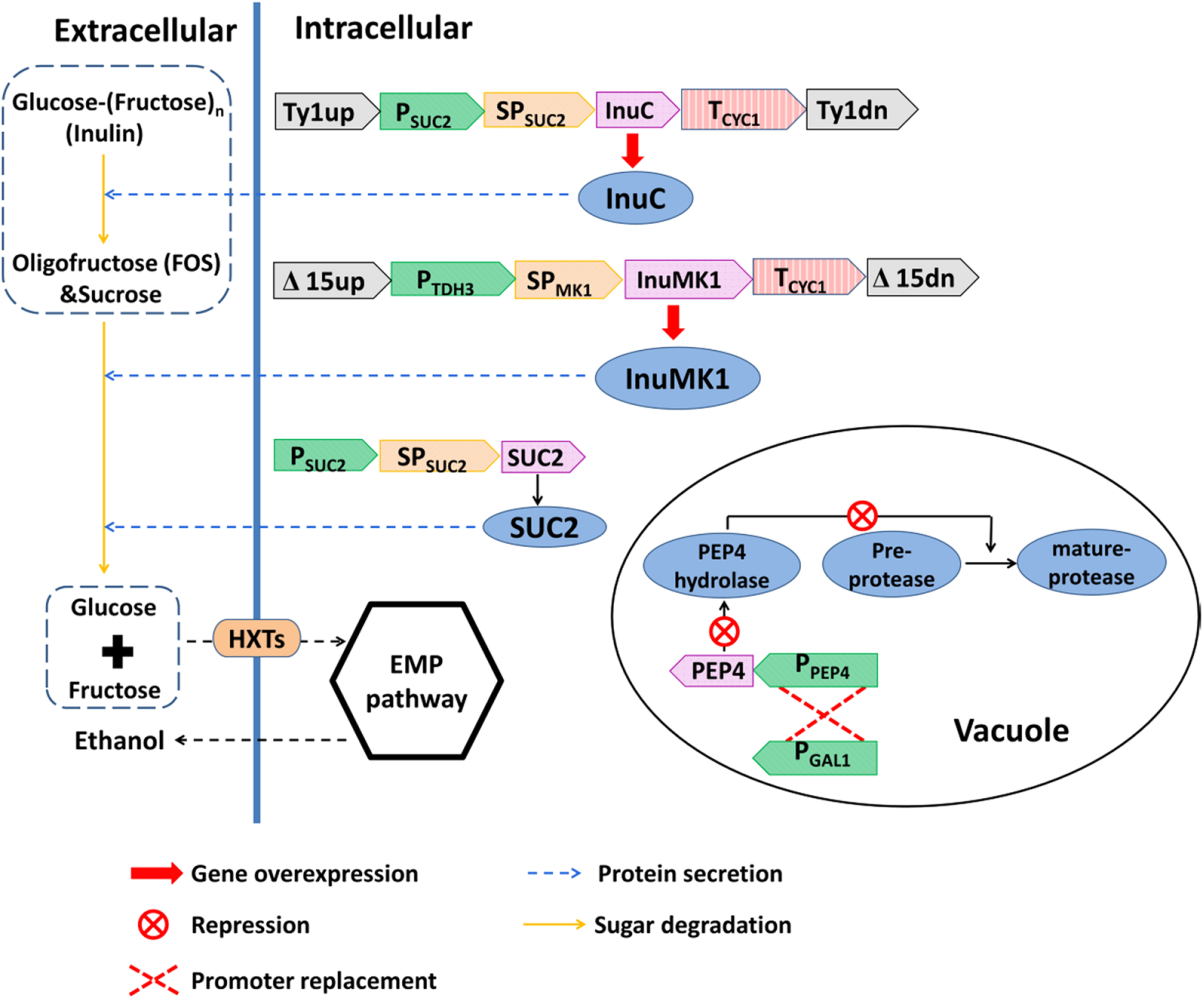
Design for conversion of inulin into ethanol in *S. cerevisiae*

Expression of heterologous exo-inulinases or endo-inulinases has been adopted to improve inulin conversion in *S. cerevisiae* [20–23]. In addition, “inulin-positive” yeast strains have been identified and evaluated in ethanol fermentation from inulin or Jerusalem artichoke tuber powder [10, 18, 24, 25]. Although these efforts improved ethanol production from inulin, ethanol productivity and yield remain to be increased. Rational engineering strategies may promote the construction of a highly efficient CBP strain for ethanol production from inulin. As shown in Fig. 1, secretive endo-inulinases can digest long-chain fructooligosaccharides into short-chain molecules that will be readily hydrolyzed into fructose and glucose by exo-inulinases. Vacuolar proteases often degrade heterologous proteins expressed in *S. cerevisiae* and thus repression of proteases may enhance the activity of heterologous inulinases.

In the present study, a natural *S. cerevisiae* isolate JZ1C was used to construct CBP strains for ethanol fermentation from inulin. Our previous study showed that inulin utilization in this strain was superior to other studied isolates and the S288C-isogenic laboratory strain BY4741, although it cannot fully utilize inulin with high degree of polymerization values [10, 19]. The superiority of the strain in inulin utilization was due to an efficient expression and secretion of the invertase SUC2 [19, 26]. In this study, rational engineering strategies were carried out to improve inulin conversion in this strain, including secretive co-expression of heterologous exo- and endo-inulinase genes, repression of a protease, and switch between haploid and diploid strains. Ethanol fermentation from both inulin and raw Jerusalem artichoke tuber powder was evaluated in the engineered strains.

## Results

### Secretive expression of endo- and exo-inulinases in JZH

**Fig. 2:**
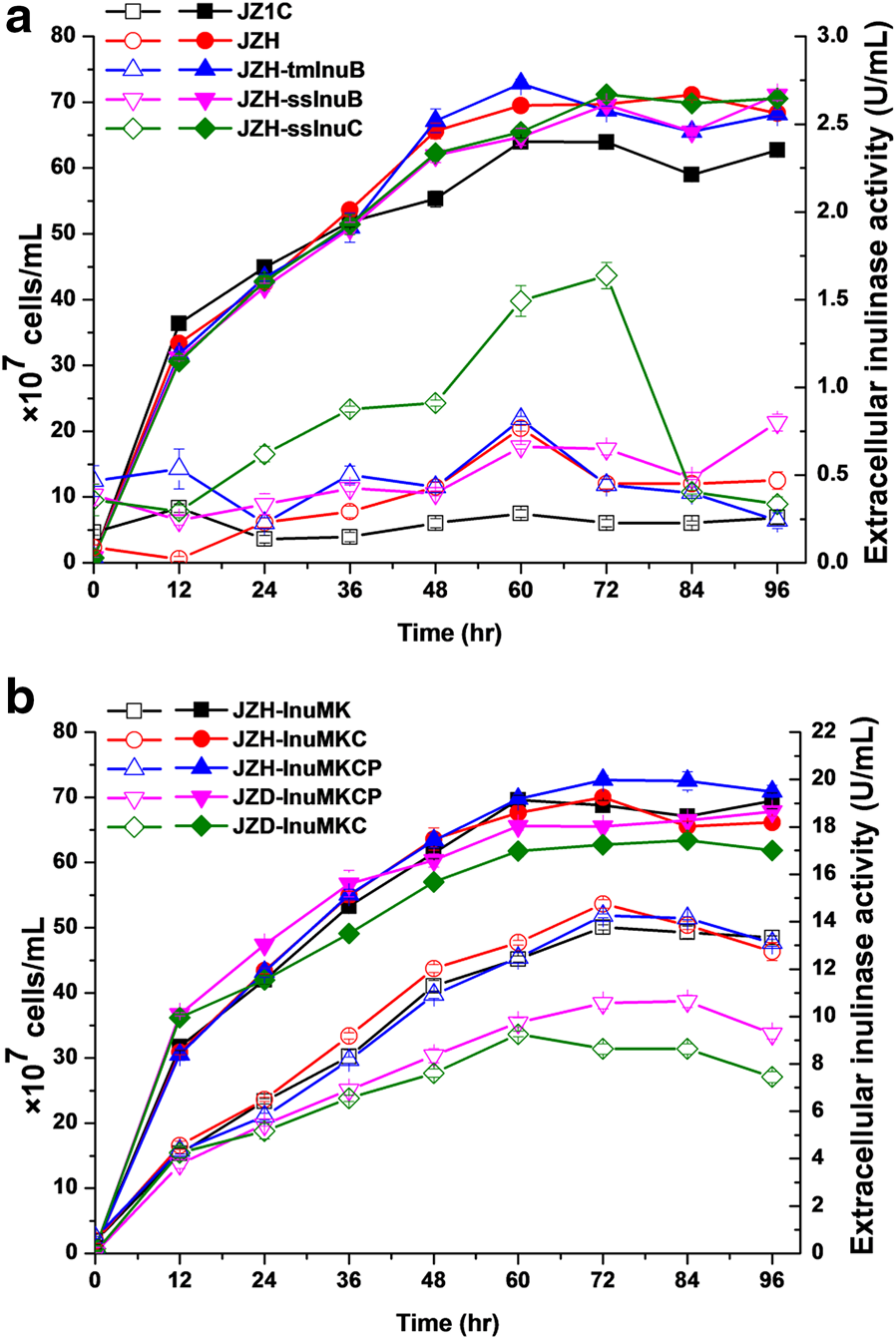
Activity dynamics of extracellular inulinase and cell growth of engineered strains. **a** Expression of heterologous endo-inulinases. **b** Comparison between haploid strains and diploid strains. *Hollow symbols* denoted extracellular inulinase activity. *Solid symbols* denoted cell density

The haploid *S. cerevisiae* strain JZH was derived from the ascospores of the diploid strain JZ1C by tetrad dissection. The endo-inulinase gene InuB of *A. niger* was integrated into the JZH genome at the loci of Ty1 under the control of the P_TDH3_ promoter and MFα signal sequence, generating a strain JZH-tmInuB (Table 1). Extracellular inulinase activity was detected during the cultivation of JZH-tmInuB in YPD medium. Unexpectedly, the inulinase activity of JZH-tmInuB was not obviously improved (Fig. 2a). To investigate the influence of signal peptide and promoter on the expression of InuB, a strain JZH-ssInuB was constructed, in which the signal sequence and the promoter of SUC2 were linked to the InuB gene. The extracellular enzyme activity dynamics of JZH-ssInuB also did not present significant differences with both JZH-tmInuB and JZH, indicating the unfeasibility of InuB in improving inulin hydrolysis in the present conditions. Afterward, a codon-optimized endo-inulinase gene InuC of *Penicillium* sp. TN-88 was synthesized and used to construct strain JZH-ssInuC. The strain JZH-ssInuC was identical to the strain JZH-ssInuB except harboring InuC instead of InuB. The strain JZH-ssInuC dramatically improved extracellular inulinase activity during cultivation between 24 and 72 h (Fig. 2a). An extracellular inulinase activity of 1.64 U/mL was achieved in the YPD culture of JZH-ssInuC at 72 h, which was 3.6-fold of that in the control strain JZH (0.45 U/mL).

**Table 1:** *Saccharomyces cerevisiae* strains used in this study

Strains	Genotype	Sources
JZ1C	Wild type, homothallic diploid	[10]
JZH	Haploid derivative of JZ1C	This study
JZH-tmInuB	*Ty1:: P*_*TDH3*_-*SP*_*MFα*_-*Inu B*-*T*_*CYC1*_	This study
JZH-ssInuB	*Ty1::P*_*SUC2*_-*SP*_*SUC2*_-*Inu B*-*T*_*CYC1*_	This study
JZH-ssInuC	*Ty1::P*_*SUC2*_-*SP*_*SUC2*_-*Inu C*_*opz*_-*T*_*CYC1*_	This study
JZH-InuMK	*YPRCΔ15::P*_*TDH3*_-*InuMK1*-*T*_*CYC1*_	This study
JZH-InuMKC	JZH-InuMK *Ty1::P*_*SUC2*_-*SP*_*SUC2*_-*Inu B*-*T*_*CYC1*_	This study
JZH-InuMKCP	JZH-InuMKC *P*_*PEP4*_ *::P*_*Gal1*_, haploid	This study
JZD-InuMKC	JZH-InuMKC *ho::HO*, diploid	This study
JZD-InuMKCP	JZH-InuMKCP *ho::HO*, diploid	This study

**Fig. 3:**
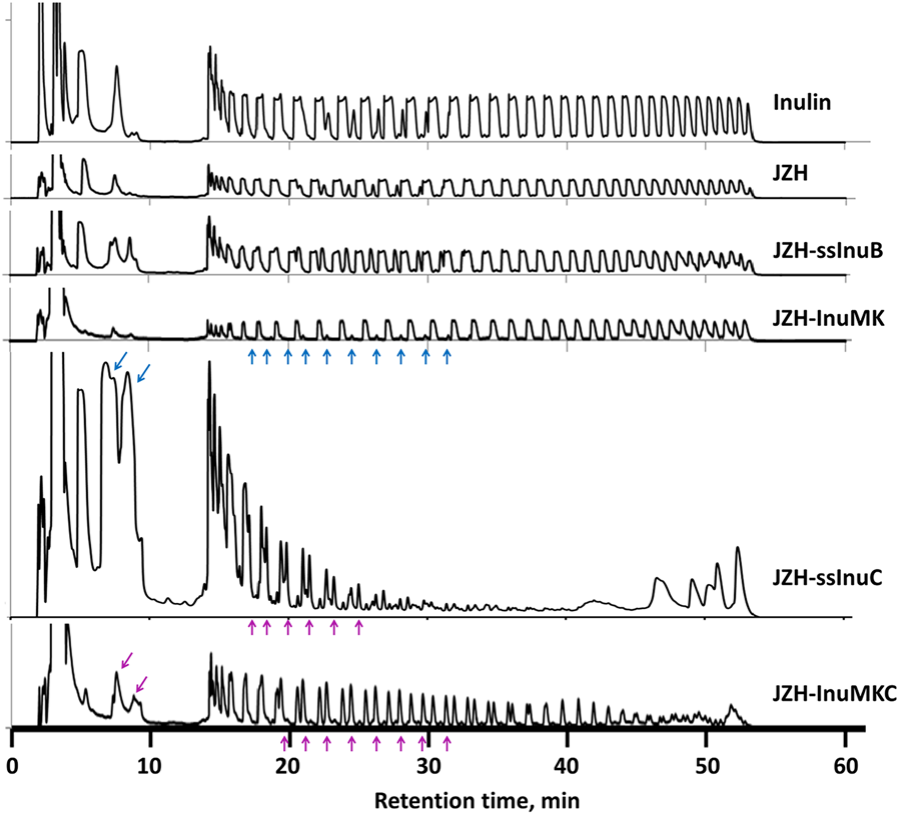
HPAEC–PAD analysis of inulin hydrolysates catalyzed by extracellular inulinase of engineered strains. The strains were cultured in YPD medium. Culture supernatant at 48 h was incubated with 2 % of inulin (w/v) for 8 h at 55 °C. The reaction mixture was analyzed by HPAEC–PAD. Inulin solution was used as the control. The *arrows* denoted the oligosaccharides hydrolyzed by exo-inulinase or generated from long-chain inulin molecules by endo-inulinase digestion

To further improve inulin utilization by *S. cerevisiae*, an exo-inulinase gene InuMK1 with inherent signal sequence from *K. marxianus* PT-1 was integrated into the loci of δ15 sites under the control of the P_TDH3_ promoter, resulting in a strain JZH-InuMK. The strain JZH-InuMK greatly increased extracellular inulinase activity during cultivation in YPD medium. An enzyme activity of 13.8 U/mL was achieved at 72 h in JZH-InuMK, showing a 30-fold increase than the control strain JZH. The strain JZH-InuMKC co-expressing the genes InuC and InuMK1 further enhanced extracellular inulinase activity to 14.8 U/mL at 72 h (Fig. 2b). HPAEC–PAD analysis of inulin hydrolysates catalyzed by extracellular inulinase of engineered strains further confirmed the efficient expression and secretion of InuC and InuMK1 (Fig. 3). The strain JZH-InuMK could efficiently utilize oligosaccharides with low and medium DP, while the strain JZH-ssInuC could hydrolyze long-chain oligosaccharides into short-chain molecules (Fig. 3). These results indicated the feasibility of the codon-optimized endo-inulinase gene InuC and the exo-inulinase gene InuMK1 in promoting inulin hydrolysis in engineered *S. cerevisiae* strains.

### Repression of the proteinase gene PEP4

Vacuolar proteases often degrade heterologous proteins expressed in *S. cerevisiae.* The proteinase gene PEP4 is among the major functional proteases, and disruption of PEP4 is able to increase heterologous protein production [27]. Considering that disruption of PEP4 may reduce strain biomass and robustness, we replaced the promoter of PEP4 using the inducible GAL1 promoter in the strain JZH-InuMKC, resulting in the strain JZH-InuMKCP. The strain JZH-InuMKCP did not display an increase in inulinase activity compared with strain JZH-InuMKC in YPD medium, which contained glucose repressing the expression of GAL1 promoter (Fig. 2b). Nevertheless, both inulinase activity and cell density decreased faster after cultivation for 72 h in JZH-InuMKC than in JZH-InuMKCP (Fig. 2b). These results indicated that repression of PEP4 might influence the cell physiology in late stationary phase.

### Inulinase activity in haploid and diploid engineered strains

**Fig. 4:**
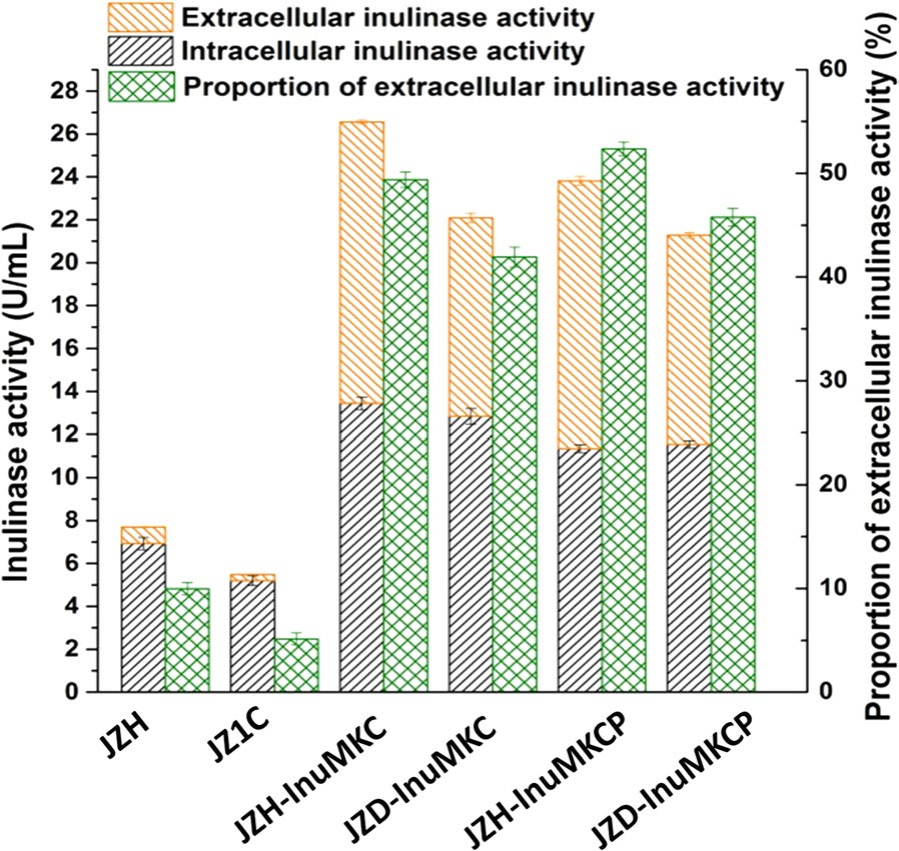
Efficiency of inulinase secretion in haploid and diploid strains inferred from extracellular and intracellular inulinase activity at 60 h during aerobic culture

Diploid strains are considered to produce enzymes more efficient than isogenic haploid strain because of harboring double heterologous genes. To test the hypothesis, the haploid engineered strains JZH-InuMKC and JZH-InuMKCP were recovered to diploid strains JZD-InuMKC and JZD-InuMKCP, respectively, by complementation of the HO gene in their genomes. The diploid state of JZD-InuMKC and JZD-InuMKCP was confirmed by morphology and sporulation observation (see Additional file 1: Fig. S1). Unexpectedly, the extracellular inulinase activity in diploid strains JZ1C, JZD-InuMKC, and JZD-InuMKCP were lower than the corresponding haploid strains JZH, JZH-InuMKC, and JZH-InuMKCP, respectively (Fig. 2b). The haploid strains presented a higher cell density than corresponding diploid strains, which might cause the difference of extracellular inulinase activity. The extracellular and intracellular inulinase activity of the engineered strains was detected at 60 h during aerobic culture. The ratio of extracellular to intracellular inulinase activity achieved in JZH-InuMKC and JZD-InuMKC was 49.4 versus 41.9 %, and that achieved in JZH-InuMKCP and JZD-InuMKCP was 52.4 versus 45.8 %, respectively (Fig. 4). The results implied that inulinase secretion in haploid strains was more efficient than the corresponding diploid strains. Haploid strains have a smaller size and a larger surface-area-to-volume ratio than the diploid strains, which was proposed favoring inulinase secretion (see Additional file 1: Fig. S1).

The PEP4-repressed diploid strain JZD-InuMKCP presented a stronger stability in inulinase activity and cell survival than JZD-InuMKC in late stationary phase (Fig. 2b). This phenomenon is identical to that found in the haploid strains JZH-InuMKC and JZH-InuMKCP. Thus, the findings in both haploid and diploid engineered strains supported that repression of PEP4 influences the cell physiology in late stationary phase.

### Ethanol fermentation from inulin

**Fig. 5:**
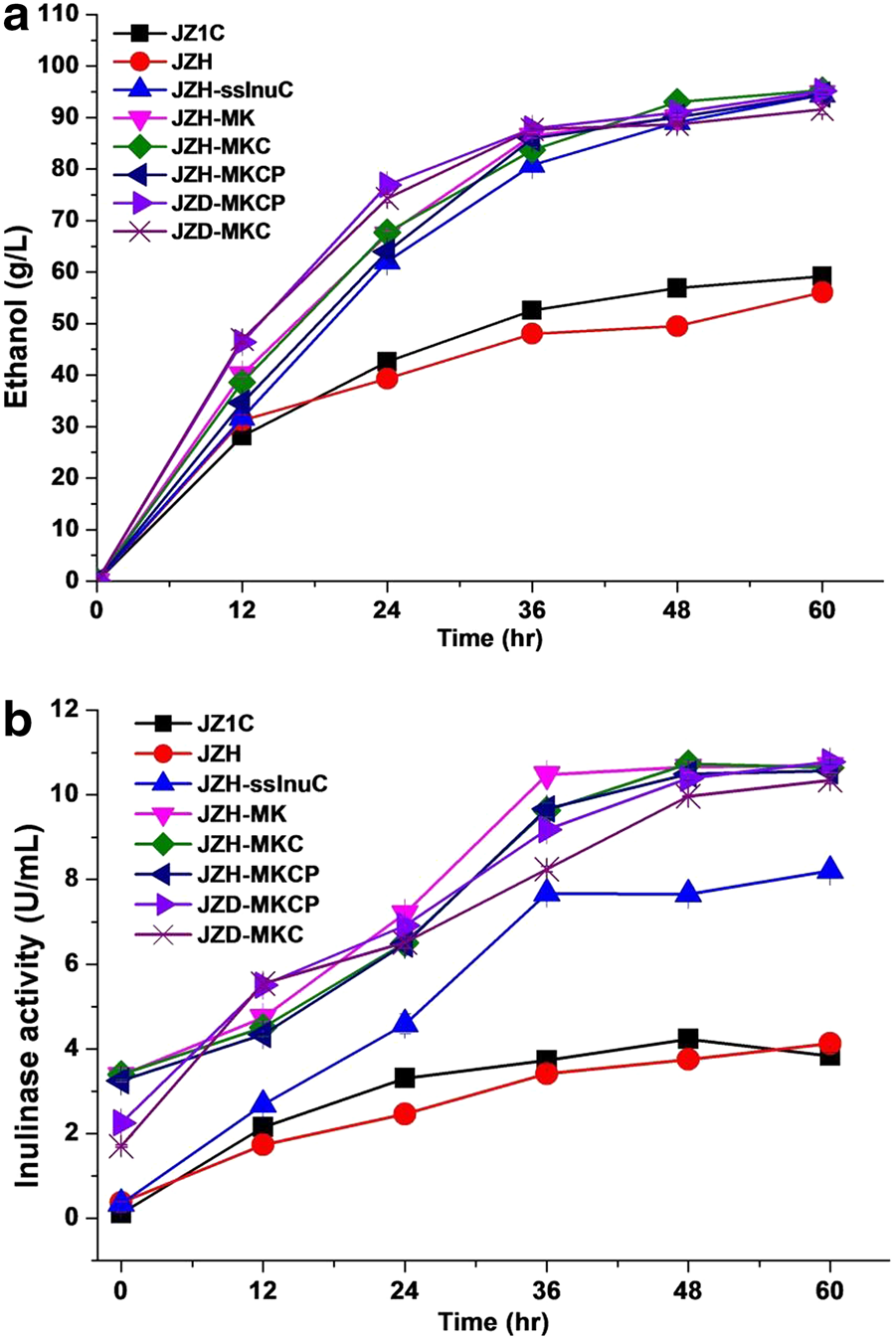
Ethanol fermentation from inulin by engineered strains. **a** Data on ethanol production. **b** Dynamics of extracellular inulinase activity during ethanol fermentation

Ethanol fermentation from inulin was performed using original and engineered strains. All strains expressing InuC and/or InuMK1 genes produced approximate 95 g/L of ethanol within 60 h from 200 g/L of inulin, corresponding to 95 % of the theoretical ethanol yield (Table 2). However, the ethanol productivity at early stage of fermentation was different among these strains. The strain JZH-ssInuC presented the lowest ethanol productivity of 2.59 g/L/h within 24 h, while a highest ethanol productivity of 3.20 g/L/h within 24 h was achieved in the diploid engineered strain JZD-InuMKCP (Table 2; Fig. 5a). Both diploid strains JZD-InuMKC and JZD-InuMKCP displayed higher ethanol productivity than the corresponding haploid strains JZH-InuMKC and JZH-InuMKCP (Table 2; Fig. 5a). Overall, the inulinase activity dynamics was consistent with ethanol production in engineered strains (Fig. 5b). The glycerol and acetic acid production maintained at a low level during ethanol fermentation and the titer of both metabolites were similar among the engineered strains (see Additional file 1: Fig. S2). Considering that the diploid strain JZD-InuMKCP displayed highest productivity within 24 h (3.20 g/L/h) and 36 h (2.44 g/L/h), this strain was used to evaluate ethanol fermentation from Jerusalem artichoke tuber powder.

**Table 2:** Data on ethanol fermentation from inulin and Jerusalem artichoke tuber powder

Strain	Ethanol productivity (g/L/h)		Maximum ethanol titer (g/L)	Of theoretical ethanol yield (%)	Yield (g/g)
	24 h	36 h			
*Inulin*					
JZ1C	1.77 ± 0.03	1.46 ± 0.01	59.16 ± 0.54	59.0	0.302
JZH	1.64 ± 0.04	1.33 ± 0.01	56.05 ± 0.29	55.9	0.286
JZH-ssInuC	2.59 ± 0.01	2.25 ± 0.01	94.44 ± 0.44	94.3	0.482
JZH-InuMK	2.81 ± 0.03	2.40 ± 0.01	94.73 ± 0.83	94.6	0.483
JZH-InuMKC	2.82 ± 0.04	2.33 ± 0.01	95.32 ± 0.35	95.1	0.486
JZH-InuMKCP	2.67 ± 0.01	2.39 ± 0.01	94.50 ± 0.34	94.3	0.482
JZD-InuMKCP	3.20 ± 0.03	2.44 ± 0.01	95.19 ± 0.13	95.0	0.486
JZD-InuMKC	3.10 ± 0.03	2.44 ± 0.03	91.60 ± 0.99	91.4	0.467
*JA powder*					
JZ1C	2.06 ± 0.00	1.50 ± 0.01	59.32 ± 0.07	66.5	0.340
JZD-InuMKCP	3.13 ± 0.01	2.17 ± 0.00	81.76 ± 0.14	91.7	0.469

### Ethanol fermentation from Jerusalem artichoke tuber powder

**Fig. 6:**
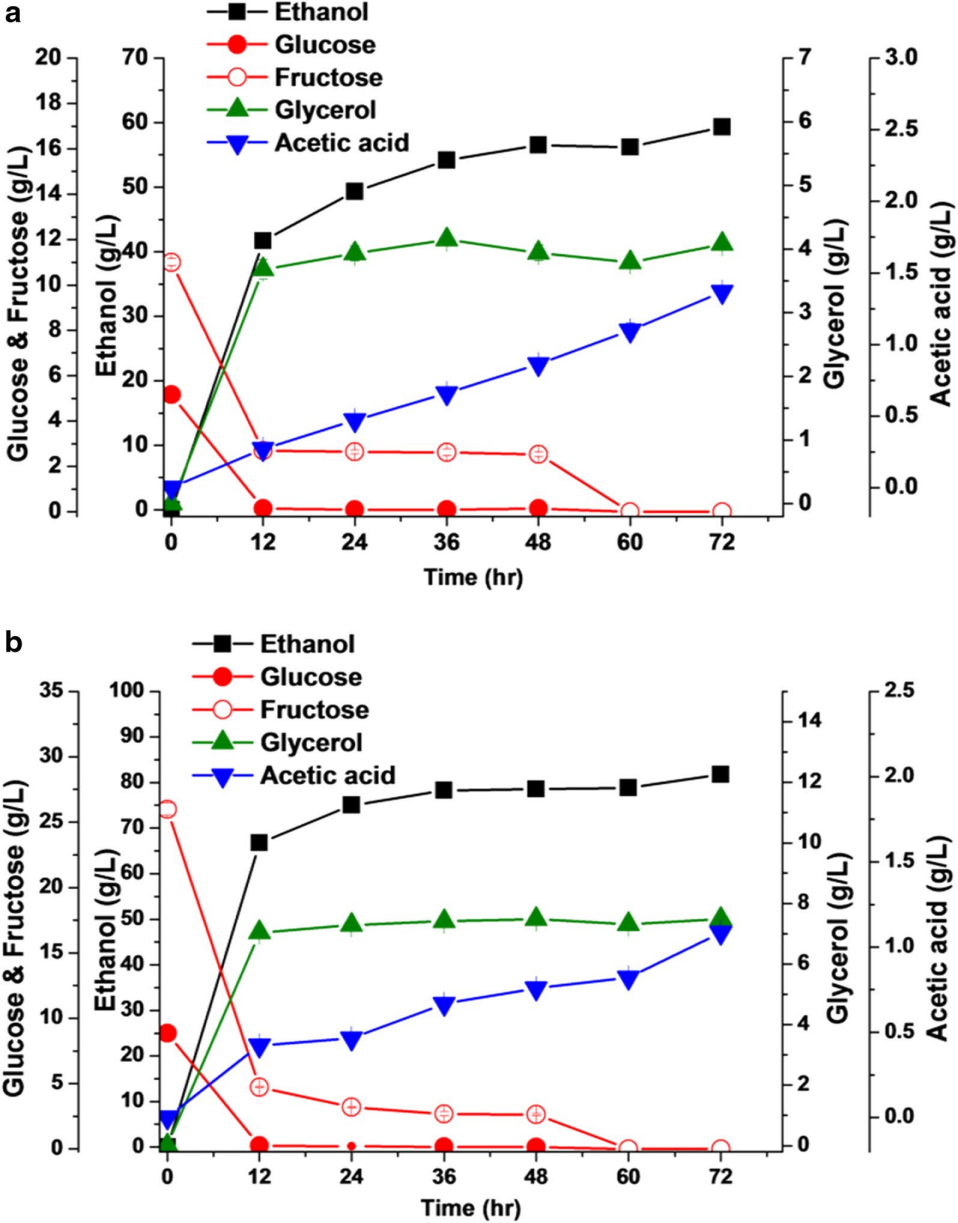
Ethanol fermentation from Jerusalem artichoke powder. **a** Data on ethanol fermentation by original strain JZ1C; **b** Data on ethanol fermentation by engineered strain JZD-InuMKCP

Ethanol fermentation from 250 g/L of Jerusalem artichoke tuber powder was evaluated using the engineered strain JZD-InuMKCP and the original strain JZ1C. The sugar content in Jerusalem artichoke tuber powder was approximately 70 % (w/w) (see Additional file 1: Table S1). The Jerusalem artichoke tuber powder used in fermentation was not exposed to thermal pretreatment and supplemented with any additional nitrogen resource. The ethanol productivity, titer, and yield achieved in the strain JZD-InuMKCP were 3.13 g/L/h (within 24 h), 81.8 g/L, and 0.469 g/g versus 2.06 g/L/h (within 24 h), 59.3 g/L, and 0.34 g/g in the strain JZ1C, respectively (Fig. 6). These results represented a significant improvement in ethanol fermentation from raw Jerusalem artichoke tuber powder by the engineered strain JZD-InuMKCP. These results also indicated that the engineered strain can efficiently perform ethanol fermentation by CBP from raw Jerusalem artichoke tuber powder without sterilization, pretreatment, and additional nutrients supplementation.

## Discussion

### Efficiency of engineered strains

This study engineered a natural *S. cerevisiae* strain to produce ethanol from a plant storage polysaccharide inulin by CBP. Secretive co-expression of exo-inulinases and endo-inulinases dramatically improved ethanol production. Repression of the protease PEP4 suspended cell death and the decrease of enzyme activity in late stationary phase. Switch haploid engineered strains to diploid strains disclosed their different properties in enzyme production and ethanol fermentation. The ultimately engineered strain JZD-InuMKCP presented significantly elevated ethanol productivity in fermentation from both inulin (2.44 g/L/h) and Jerusalem artichoke tuber powder (3.13 g/L/h) (Table 2). To our knowledge, these are the highest productivities reported up to now in ethanol fermentation from inulin resources [22, 23, 28]. Ethanol yield achieved is close to the theoretical maximum in most engineered strains (Table 2). In our study, 250 g/L of Jerusalem artichoke tuber powder was used in ethanol fermentation. The Jerusalem artichoke tuber powder at a concentration of 300 g/L absorbed much water and formed semi-solid, which hindered ethanol fermentation.

### Ploidy of engineered strains and ethanol fermentation

Most natural *S. cerevisiae* strains are homothallic diploids, while haploid strains are preferred in bioengineering [29, 30]. In this study, the haploid strain JZH dissected from diploid natural strain JZ1C was used as the starting host. The engineered haploid strains were ultimately switched to diploid strains. The isogenic haploid and diploid strains presented similar ethanol titer and yield, though the extracellular inulinase activity in haploid strains is higher than that in their diploid counterparts (Fig. 2). However, the diploid engineered strains displayed higher ethanol productivity during ethanol fermentation from inulin (Fig. 5). A recent study reported that diploid *S. cerevisiae* strains hybridized from haploids improved the xylose fermenting capability due to doubling exogenous xylose-assimilating pathway [31]. However, another study did not observe the benefits of an increased ploidy for xylose metabolism in their engineered *S. cerevisiae* strains [32]. Instead, they found that the improvement in xylose fermentation by hybrid diploid strains was because of the complementation of two discrepant xylose-metabolic pathways, but not genome duplication [32]. In this study, we found that the haploid strains exhibited superiority in production and secretion of heterologous enzymes, while the diploid strains presented advantages in ethanol fermentation (Figs. 2, 5). The advantages of haploid strains in production and secretion of heterologous enzymes were attributed to larger cell density and surface-area-to-volume ratio than the diploid strains. The detection of inulinase activity did not explain the advantage of diploid strains in ethanol fermentation productivity. Double inherent enzyme genes in diploid strains may benefit ethanol fermentation, such as enzyme genes in the EMP pathway.

### Natural strains used as bioengineering hosts

Either model microorganisms or natural strains are like a coin having two sides. It is not easy to conclude whether model microorganisms or natural strains are prior hosts for bioengineering [33]. Model strains have extensively been used as bioengineering hosts because of clear background knowledge and available genetic tools. However, some natural strains have excellent properties in substrate utilization and stress tolerance. For example, the natural *S. cerevisiae* strain JZ1C used in this study possesses advantages in inulin utilization and heat tolerance [10, 30]. Secretive expression of heterologous inulinase genes in strain JZ1C further dramatically improved inulin utilization and ethanol fermentation. Population genetics and ecology studies disclosed a panel of *S. cerevisiae* strains owing priorities to model strains in tolerance to specific stressors and utilization of particular carbon sources [8, 30, 34–37]. Although genetic manipulation for natural strains in many cases is not as easy as model strains, it is feasible for the most extensively used organisms *S. cerevisiae* and *E. coli*. The Cre-loxP or FLP-FRT marker rescue system can be easily used in natural *S. cerevisiae* and *E. coli* strains avoiding screening of auxotrophs [38, 39]. Recent studies on natural populations and experimental evolution of *E. coli* identified strains exhibiting special properties, including tolerance to environmental stresses, utilization of particular carbon sources such as galactose, and low toxicity [40, 41]. Based on this study and the background knowledge, we suggested that specific strains of model microorganisms can be used as bioengineering hosts for special applications.

## Conclusions

In this study, we engineered a natural yeast strain for ethanol fermentation from inulin by secretive co-expression of inulinases, repression of a protease, and switch of strain ploidy. The haploid engineering strains exhibited superiority in production and secretion of heterologous enzymes, while the diploid engineering strains presented advantages in ethanol fermentation. Highly efficient ethanol fermentation from both inulin and Jerusalem artichoke tuber powder was achieved in the ultimate diploid strain JZD-InuMKCP, which indicated the feasibility of natural strains used as bioengineering hosts. The findings in this study provided guidelines for host selection in bioengineering.

## Methods

### Strains and media

Strains used in this study are listed in Table 1. Yeast strains were routinely cultured at 30 °C in YPD medium (10 g/L yeast extract, 20 g/L peptone, 20 g/L glucose). The antibiotics G418 or Zeocin were added to YPD medium at a final concentration of 400 mg/L when yeast transformants were screened. YPG broth (10 g/L yeast extract, 20 g/L peptone, 20 g/L galactose) was employed to induce the expression of Cre recombinase for the rescue of antibiotics. Sporulation of *S. cerevisiae* strains was induced in McClary media (1 g/L Glucose, 1.8 g/L KCl, 8.2 g/L NaAc, 2.5 g/L yeast extract, and 15 g/L agar powder). *E. coli* was grown in Luria–Bertani medium. Ampicillin (100 mg/L) was added to the medium when required.

### Dissection of the haploid strain JZH

The *S. cerevisiae* strain JZ1C is a homothallic diploid [19]. Haploid ascospores of JZ1C are capable of switching mating type by expressing the HO endonuclease and selfing to restore a diploid state. Stable haploid ascospores were obtained by disruption of an HO allele in JZ1C and subsequent sporulation and tetrad dissection. The expected 2:2 pattern of segregation of HO and HO::kanMX genes was observed on yeast YPD and YPD plus G418 plates. A haploid strain JZH was derived from one of the ascospores harboring HO::kanMX by rescue of the selective marker. The haploid state of JZH was confirmed by diagnostic PCR and sporulation observation (see Additional file 1: Fig. S1). Cells of JZH displayed a smaller size compared with its diploid parent JZ1C and tended to gather into clumps when cultivated in YPD (see Additional file 1: Fig. S1). The variations of cell size between JZ1C and JZH resulted in a different ratio of optical density (OD_600_) to cell density. One OD_600_ of JZ1C and JZH culture corresponded to a cell density of 3.32 ± 0.05 × 10^7^ and 3.73 ± 0.07 × 10^7^ cells/mL, respectively. The haploid strain JZH was used as the initial host organism.

### Molecular biology techniques

Yeast genomic DNA was extracted using E.Z.N.A.^®^ Yeast DNA Kit (Omega Bio-Tek Inc., GA, USA). PCR amplification was performed with high fidelity DNA polymerase PrimeSTAR^®^ HS (Takara, Japan). Diagnostic PCR amplification employed routine Taq DNA polymerase. DNA fragments were purified by E.Z.N.A.^®^ Gel Extraction Kit (Omega) or E.Z.N.A.^®^ Cycle Pure Kit (Omega). Plasmids were isolated from *E. coli* with E.Z.N.A.^®^ Plasmid Mini Kit I (Omega).

### Plasmid construction

Plasmids used in this study are summarized in Table 3. Gibson assembly cloning kit (New England Biolabs) was used to construct plasmids. The endo-inulinase gene InuB and the exo-inulinase gene InuMK1 were amplified from *Aspergillus niger* and *Kluyveromyces marxianus*, respectively [42, 43]. Coding sequences of an endo-inulinase gene in *Penicillium* sp. TN-88 were codon-optimized for *S. cerevisiae* and synthesized by Beijing Awigene Technology (Beijing, China) (see Additional file 1: Fig. S3). These heterologous genes were flanked by *S. cerevisiae* promoters *P*_*TDH3*_ or *P*_*SUC2*_ and the terminator *T*_*CYC1*_. The signal sequences of *S. cerevisiae* mating factor α (MFα), invertase (SUC2), or *K. marxianus* inulinase (INU1) were used to induce protein secretion. Ty1 retrotransposons and delta sequences *YPRCΔ15* were employed as genomic integration sites of heterologous gene cassettes.

**Table 3:** Plasmids constructed and used in this study

Plasmids	Characteristics	Sources
pMD19-T	Delivery vectors	Takara
pUG6	Backbone vectors	EUROSCARF
pSH47	GAL1 promoter	EUROSCARF
pSH65	*Cre* recombinase gene, Zeocin	EUROSCARF
pUG6-tmInuB	*Ty1*-*P*_*TDH3*_-*SP*_*MFα*_-*InuB*-*T*_*CYC1*_	This study
pUG6-ssInuB	*Ty1*-*P*_*SUC2*_-*SP*_*SUC2*_-*InuB*-*T*_*CYC1*_	This study
pUG6-ssInuC	*Ty1*-*P*_*SUC2*_-*SP*_*SUC2*_-*InuC*_*opz*_-*T*_*CYC1*_	This study
pUG6-PT	*P*_*TDH3*_-*T*_*CYC1*_	This study
pUG6-InuMK	*P*_*TDH3*_-*InuMK1*-*T*_*CYC1*_	This study
pUG6-PEP4	*P*_*GAL1*_-*PEP4*	This study
pUG6-HOre	*HO* cassette	This study
pUG6-HOdel	Cassette for deletion of the *HO* gene	This study
pUC-InuC_**opz**_	Codon-optimized *InuC* gene	This study

### Strain construction

The *S. cerevisiae* strains described in this study were all derived from the wild-type diploid strain JZ1C [10]. Yeast strains were transformed by lithium acetate method [44]. To obtain the haploid strain JZH, the *HO* alleles in strain JZ1C were disrupted before ascospores were induced and dissected. Ascospores were dissected by using Narishige micromanipulators (Narishige International Limited, London, UK). The selective markers were looped out by transformation with the pSH65 plasmid, expressing the inducible Cre recombinase and carrying the phleomycin resistance gene *ble*^*r*^ [39]. Loss of pSH65 was achieved by growing cells in liquid YPD medium at 30 °C for 2 days. The cassettes for transforming strain JZH were amplified from plasmids pUG6-tmInuB, pUG6- ssInuB, pUG6- ssInuC, and pUG6- InuMK (see Additional file 1: Fig. S4; Table 3). To replace the promoter of PEP4 gene, the transforming cassette was amplified from the plasmid pUG6-PEP4. The diploid transformants JZD-InuMKC and JZD-InuMKCP were derived from the haploid transformants JZH-InuMKC and JZH-InuMKCP by complementing the *HO* gene, respectively (Table 1). In all cases gene integration, promoter replacement, and marker rescue were confirmed by diagnostic PCR.

### Inulinase activity assay

Inulinase activity was assayed by determining the concentration of released reducing sugars according to Hu et al. [10] with minor modification. One unit of inulinase activity was defined as the amount of enzyme that produces 1 μmol fructose per min under the assay conditions. The reaction mixture containing 50 μL of culture supernatant and 450 μL of 2 % inulin (dissolved in 0.1 M acetate buffer, pH 5.0) was incubated at 50 °C for 15 min. The reaction was terminated at 100 °C for 10 min, and the concentration of reducing sugar in the mixture was determined [45]. Absorbance was read at a wavelength of 540 nm. To detect intracellular inulinase activity, yeast cells harvested from 1 mL culture were washed and resuspended into 2 mL sodium acetate buffer (pH 5.4) supplemented with 1/100 (v/v) protease inhibitor cocktail (Ameresco). Afterward, the yeast cells were disrupted at 35 kpsi by three shots using a cell disruptor (Constant Systems Ltd.). Next, the disrupted cell suspension was centrifuged at 10,000*g* for 5 min, and the supernatant was used for intracellular inulinase activity assay.

### Ethanol fermentation

Ethanol fermentation from inulin and Jerusalem artichoke tuber powder (JAP) was performed using YPI and JAP media, respectively. The content of hexose in inulin and Jerusalem artichoke tuber powder detected by HPLC after acid pretreatment is shown in Additional file 1: Table S1. The YPI fermentation medium consists of 10 g/L yeast extract, 20 g/L peptone, and 200 g/L chicory inulin. The JAP fermentation medium contains 250 g/L Jerusalem artichoke tuber powder without any other nutrient supplements. The Jerusalem artichoke tubers were purchased from a local market in Qingdao, China. Preparation of the tuber flour was performed as described in our previous study [10]. Sterilization was employed for YPI media at 115 °C for 20 min but not for JAP media. The anti-foam agent T-F (0.3 %, v/v) was added before starting fermentation. The strains used in fermentation were recovered in YPD medium from stocks at −80 °C and then they were cultivated in 50 mL YPD seed culture to a cell density of approximate 7.5 × 10^6^ cells/mL. The seed culture was inoculated at a size of 10 % (v/v) into 72 mL YPI or JAP media loaded in 100 mL flasks. The fermentation was performed at 30 °C with a shake at 100 rpm. Samples were taken at an interval of 12 h and centrifuged at 10,000 rpm for 5 min to separate the supernatant, which was stored at −80 °C for inulinase activity assay and HPLC detection.

### Analytical methods

Culture supernatants and media were filtered with 0.22 μm hydrophilic filters after centrifugation. The components of filtered solutions glucose, fructose, ethanol, glycerol, and acetic acid were detected by Agilent HPLC using a Hi-Plex H column (300 × 7.7 mm) operated at 55 °C with 5 mM H_2_SO_4_ as mobile phase at a flow rate of 0.5 mL/min. A refractive index detector (RID) was used at 35 °C in HPLC detection.

The total hexose sugars in inulin and Jerusalem artichoke tuber powder were estimated by HPLC after acid hydrolysis (see Additional file 1: Table S1). In acid hydrolysis process, inulin or Jerusalem artichoke tuber powder (0.25 g) was dissolved in 72.5 mL of 4 % (w/w) H_2_SO_4_ and boiled for 10 min. The reaction was terminated by cooling in ice water. Three milliliter of hydrolysate was neutralized to pH 6.0–7.0 using CaCO_3_ and then centrifuged at 10,000*g* for 5 min. The supernatants were filtered with 0.22 μm filter and analyzed by HPLC.

The inulin-type oligosaccharides produced in inulin fermentation by yeast strains were detected using high-performance anion-exchange chromatography with pulsed amperometric detection (HPAE-PAD) as described in our previous study [10].

## Additional file

10.1186/s13068-016-0511-4 The content of hexose in inulin and Jerusalem artichoke tuber powder (JAP). **Fig. S1**. Cell morphology and sporulation observed by optical microscopy. **Fig. S2**. Data on ethanol fermentation from inulin. **Fig. S3**. Codon-optimized sequences of the endo-inulinase gene in *Penicillium* sp. TN-88. **Fig. S4**. DNA cassettes used for transformation of *S. cerevisiae*

## Abbreviations

CBP: consolidated bioprocessing
DP: degree of polymerization
JAP: Jerusalem artichoke tuber powder
EMP: Embden–Meyerhof–Parnas pathway
RID: refractive index detector
HPAE-PAD: high-performance anion-exchange chromatography with pulsed amperometric detection
