# Supplementary material for: Engineering a natural Saccharomyces cerevisiae strain for ethanol production from inulin by consolidated bioprocessing
Source: Biotechnol Biofuels. 2016 Apr 30;9:96. doi: 10.1186/s13068-016-0511-4 (PMC4851821; doi:10.1186/s13068-016-0511-4)
Supplement: Supplementary file 1 — 10.1186/s13068-016-0511-4 The content of hexose in inulin and Jerusalem artichoke tuber powder (JAP). Fig. S1. Cell morphology and sporulation observed by optical microscopy. Fig. S2. Data on ethanol fermentation from inulin. Fig. S3. Codon-optimized sequences of the endo-inulinase gene in Penicillium sp. TN-88. Fig. S4. DNA cassettes used for transformation of S. cerevisiae [file 13068_2016_511_MOESM1_ESM.pdf]

Supplementary materials

Engineering a natural *Saccharomyces cerevisiae* strain for ethanol production from inulin by consolidated bioprocessing

Da Wang[1,3], Fu-Li Li*[1, 2], Shi-An Wang*[1,2]

1. Shandong Provincial Key Laboratory of Synthetic Biology, Qingdao Institute of Bioenergy and Bioprocess Technology, Chinese Academy of Sciences, Qingdao, 266101, China.

2. Key Laboratory of Biofuels, Qingdao Institute of Bioenergy and Bioprocess Technology, Chinese Academy of Sciences, Qingdao, 266101, China.

3. University of Chinese Academy of Sciences, Beijing 100039, China.

*Corresponding author:

Shi-An Wang, Tel.: +86-532-80662656; Fax: +86-532-80662778.

E-mail: wangsa@qibebt.ac.cn

Fu-Li Li, Tel.: +86-532-80662655; Fax: +86-532-80662778.

E-mail: lifl@qibebt.ac.cn

Supplementary Figure and Table Legends

Table S1. The content of hexose in inulin and Jerusalem artichoke tuber powder (JAP).

Fig. S1. Cell morphology and sporulation observed by optical microscopy. (A) JZH, (B) JZ1C, (D) JZH-InuMKC, (E) JZD-InuMKC, (G) JZH-InuMKCP, (H) JZD-InuMKCP. (C) (F) and (I) depicted asci formation in diploid strains JZ1C, JZD-InuMKC, and JZD-InuMKCP, respectively. No asci were observed in strains JZH, JZH-InuMKC, and JZH-InuMKCP (Pictures not shown). Bar=20 μm.

Fig. S2. Data on ethanol fermentation from inulin. (A) Glucose production and assumption. (B) Fructose production and assumption. (C) Glycerol production. (D) Acetic acid production.

Fig. S3. Codon-optimized sequences of the endo-inulinase gene in *Penicillium* sp. TN-88.

Fig. S4. DNA cassettes used for transformation of *S. cerevisiae*. (A) Cassettes for overexpression of InuB gene, (B) Cassettes for overexpression of genes InuB or $InuC_{opz}$, (C) Cassettes for overexpression of InuMK1 gene, (D) Cassettes for restore of HO gene, (E) Cassettes for deletion of HO gene, (F) Cassettes for replacement of PEP4 gene promoter by GAL1 promoter.

Table S1. The content of hexose in inulin and Jerusalem artichoke tuber powder (JAP).

Feedstock	Hexose content (%)			F/G
	Glucose	Fructose	Total hexose (G+F)	
Inulin	9.79±0.14	88.22±0.14	98.01±0.06	9.01±0.13
JAP	8.78±0.20	60.99±0.38	69.77±0.57	6.95±0.11

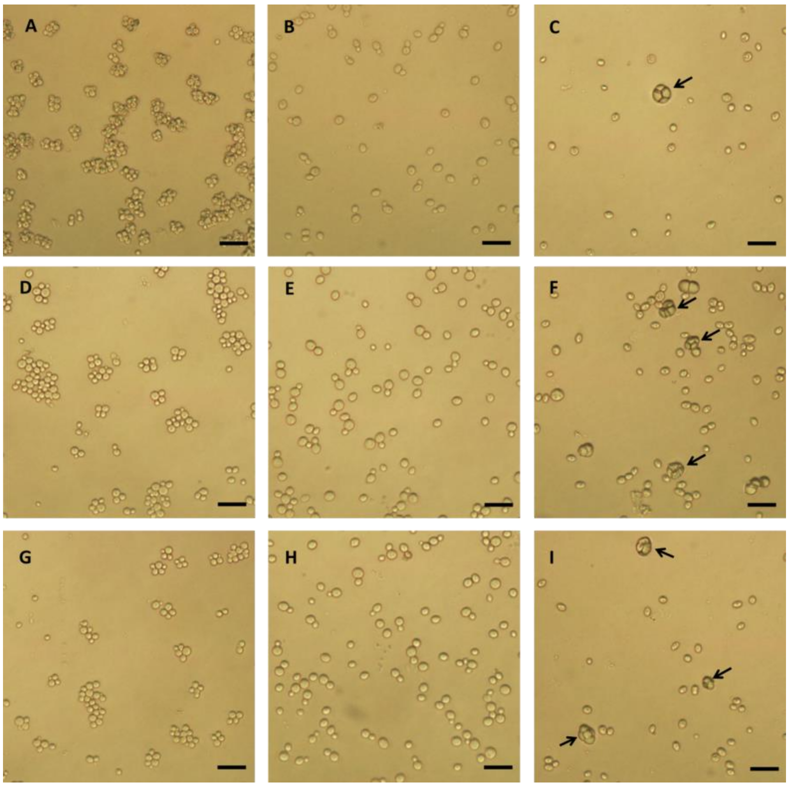

Fig. S1. Cell morphology and sporulation observed by optical microscopy. (A) JZH, (B) JZ1C, (D) JZH-InuMKC, (E) JZD-InuMKC, (G) JZH-InuMKCP, (H) JZD-InuMKCP. (C) (F) and (I) depicted asci formation in diploid strains JZ1C, JZD-InuMKC, and JZD-InuMKCP, respectively. No asci were observed in strains JZH, JZH-InuMKC, and JZH-InuMKCP (Pictures not shown). Bar=20 μm.

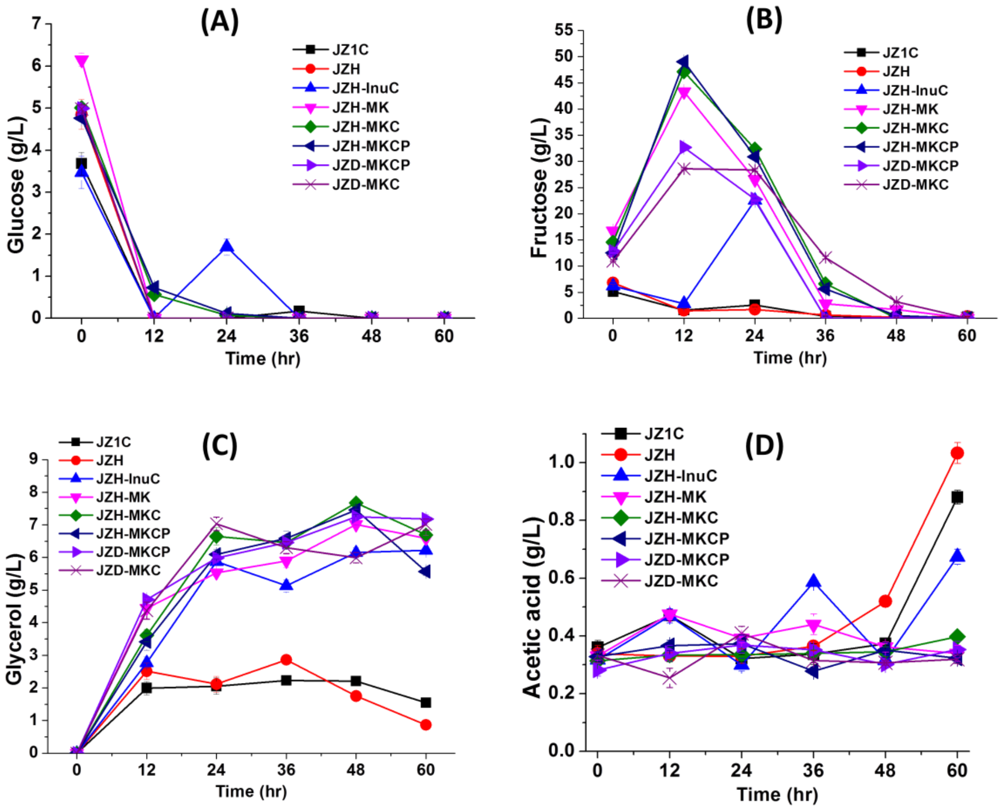

Fig. S2. Data on ethanol fermentation from inulin. (A) Glucose production and assumption. (B) Fructose production and assumption. (C) Glycerol production. (D) Acetic acid production.

1 ATGATTTCTC AGGGTTTGAC AGGTGCATTG AAGGCATTGC CATTGGTTTG CGCTTTAGTT

61 GCTAGAGCTG TTGCCGATGA CTATAGACCT GCTTTCCATT TTTGCCCAGC TGAGAATTGG

121 ATGAACGAAC CAAATGGATT AATACAGATT AACTCAACCT GGCACTTATT TTACCAAGCC

181 GATCCAGCCG CTAATGTTTG GGGTAATGAA TGTTGGGGAC ATGCTACATC TTCAGATTTA

241 TTGCACTGGG ATCATTTGCC TGTAGCTATT CCAGTCGAAA ATGGTATTGA ATCTTTTACT

301 GGTACCTCAT ATTATGATTC AAACAATACT TCTGGATTGG GTACTTCTAC TAATCCTCCA

361 TATTTAGCTT TTTTCACTGG TTATACTGAG TCTAATAAGA CTCAAGATCA AAGATTAGCA

421 TATTCAACAG ATTTGGGTCA AACATGGGTT AAATTCGCTG GTAACCCAAT AATTGGAGCT

481 GCACAAGAGG CTCCACAAGA CATCTCTGGT GGTTTGGAAT CTAGAGATCC TAAAGTATTC

541 TTTCATGCTC CATCAGGTAA ATGGGTCATG GTCTTGGCAC ATGGTGGTCA AGATAAGTTG

601 ACCTTTTGGA CCTCTTTGGA TGCTAAAAAT TGGACTTGGG TCTCTGATTT GTCATCATCT

661 CAAATAGAAG GTTTTCCTTC TTCAATTACT GGATGGGAAG TTCCAGATAT GTTTCAATTG

721 CCAATTCAAG GTATTAAGAA AACTACCTGG GTTTTGATAT TTACTCCTGC TCAAGGTTCT

781 CCACCAGGTG GTAATGGTGT TGTTGCCTTA ACTGGTTCAT TTGATGGTGA AACCTTTGTA

841 GCTGACCCAG TAGATCCATC AACTTTGTGG TTGGATTATG GTAGAGATTT CGATGGTGCC

901 TTGTCTTGGG AAAATGTTCC AGCTTCTGAC GGTAGAAGGA TAATTGCAGC AGTTATGAAC

961 TCATATGGTT CAAATCCACC AACTACAACT TGGAAAGGTA TGTTGTCATT CCCTAGAACT

1021 TTGGCTTTAA AACAAATCGG TTCTAAACAG TATTTTTTAC AACAACCTGT TGCAGAATTG

1081 TCTACTATTG ACGGTTCTTT GACATCTATC CAAAACCAGA CTATTACTCC AAATCAAACC

1141 TTATTATCAT CAATTCACGG TACATCATTG GACATTAGAA TGGCCTTCGT CATTGATTCA

1201 GGTGCTACTT TATCATTAGC TGTTAGAAAA GGTGGTTCAG AACAAACTGT TATAAGATAC

1261 TTCCAATCTA ATTCTACATT GTCTGTAGAT AGAACTGCCT CAGGTGACAT TTCTTATGAC

1321 CCTGCTGCCG GAGGAGTACA TACTGCTCAG TTGGCTCAGG ATAACACCGA ATTAGTACAC

1381 ATCTGGGCAT TGATAGATAC ATGTTCAGTT GAAGTTTTTG GTGGTGAAGG TGAAGCCGTT

1441 ATTTCTGACT TGATTTTTCC ATCTAACTCA TCTGATGGTT TGTCTTTAGA AGTTTCTGGA

1501 GGTACTGCTA TGTTGAGATC TGTTAACGTC TCTTCAGTTT CTTTGTAA

Fig. S3. Codon-optimized sequences of the endo-inulinase gene in *Penicillium* sp. TN-88.

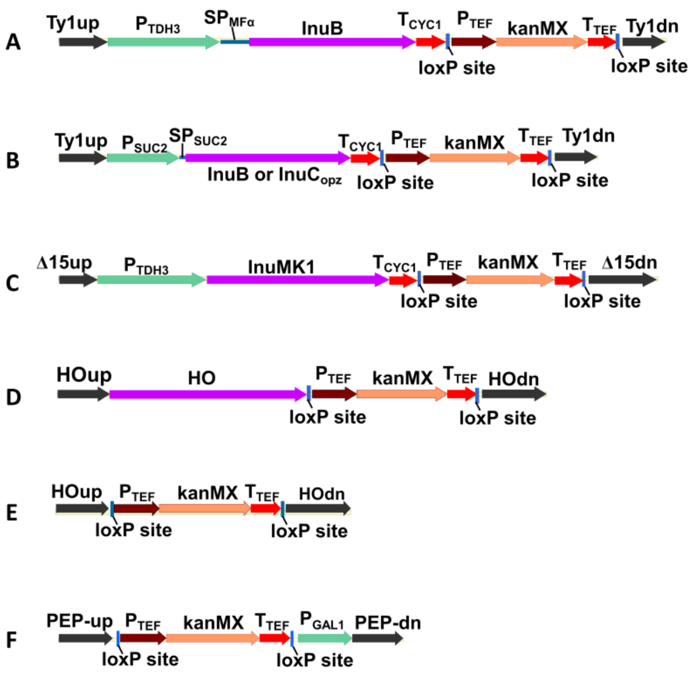

Fig. S4. DNA cassettes used for transformation of *S. cerevisiae*. (A) Cassettes for overexpression of InuB gene, (B) Cassettes for overexpression of genes InuB or InuC$_{opz}$, (C) Cassettes for overexpression of InuMK1 gene, (D) Cassettes for restore of HO gene, (E) Cassettes for deletion of HO gene, (F) Cassettes for replacement of PEP4 gene promoter by GAL1 promoter.
